# Annotated Whole-Genome Shotgun Sequence of Multidrug-Resistant *Mycobacterium tuberculosis* MTB13_M Isolated from Morocco

**DOI:** 10.1128/genomeA.01756-16

**Published:** 2017-03-02

**Authors:** L. Lahlou, N. El Mrimar, T. Alouane, M. Laamarti, S. Karti, O. Benhrif, H. El Mesbahi, H. Lemriss, F. Bssaibis, A. Maleb, S. El Rarit, A. Zegmout, R. El Jaoudi, M. Frikh, A. Lemnouar, T. Dakka, M. Elouennass, A. Ibrahimi

**Affiliations:** a Biotechnology Lab (MedBiotech), Rabat Medical & Pharmacy School, Mohammed V University, Rabat, Morocco; b Service de Bactériologie, Hôpital Militaire d’Instruction Mohammed V, Equipe d’Épidémiologie et Résistance Bactérienne (ERB), Rabat Medical & Pharmacy School, Mohammed V University, Rabat, Morocco

## Abstract

Here, we describe the annotated genome sequence of *Mycobacterium tuberculosis* MTB13_M. The organism was isolated from a sputum sample in Morocco.

## GENOME ANNOUNCEMENT

Tuberculosis (TB) is an endemic and major public health problem in Morocco (1). TB is an airborne bacterial infection caused by *Mycobacterium tuberculosis* and can be acquired by breathing contaminated air droplets coughed or sneezed by a person nearby who has active TB.

In this report, a clinical isolate of *M. tuberculosis* MTB13_M from a male patient was subjected to whole-genome shotgun sequencing.

MTB13_M was cultured in Lowenstein-Jensen culture media and its total genomic DNA was extracted using a GenoLyse kit (Hain Lifescience). DNA concentrations were determined using the NanoVuePlus spectrophotometer (Biochrom) and 1 ng of DNA was used to sequence the whole-genome of the strain. Shotgun libraries were prepared from the extracted genomic DNA following the Nextera XT protocol (Illumina) and the MiSeq-Illumina platform was used for the whole-genome sequencing.

The genome sequence of *M. tuberculosis* MTB13_M strain was determined by high-throughput sequencing performed on the genome sequencer Miseq (Illumina/Miseq) with a paired-end 250-bp sequencing kit. This platform provides smaller genome sequencing for short read lengths than other sequencing platforms to obtain raw sequences. *De novo* assemblies were performed using Spades (version 3.9) (2), with k-mers of 21, 33, 55, 77, 99, and 127 available in the Illumina genomic cloud computing platform BaseSpace (https://basespace.illumina.com). This resulted in 728,768 reads with an average read length of 250 bp and approximately 24-fold coverage. Low-quality reads/bases were filtered using Trimmomatic version 0.36 (3).

The *de novo* assembled reads generated an *N*_50_ of 25,719 bp with a genome containing a total of 4,347,307 bp, and 292 contigs with a G+C content of 65.4%.

The genome was annotated using the Rapid Annotations using Subsystems Technology (RAST) server (4, 5) for predicting subsystems, RNAs, and coding sequences (CDSs). To predict genomic objects such as CDSs and RNA genes, we used the MicroScope platform (6, 7).

The genome was identified as having 4,680 CDSs, and 45 tRNAs, and four types of rRNA (5S, 16S, and 23S) were annotated, and each has one rRNA except for type 16S that has two rRNAs. RAST identified 398 subsystems.

### Accession number(s).

This whole-genome shotgun project has been deposited at DDBJ/EMBL/GenBank under the accession number FTKO01000000. The versions described in this paper are the first versions, under Bioproject designation no. PRJEB14778.
